# Dysregulation of epithelial ion transport and neurochemical changes in the colon of a parkinsonian primate

**DOI:** 10.1038/s41531-020-00150-x

**Published:** 2021-01-21

**Authors:** Erika Coletto, Iain R. Tough, Sara Pritchard, Atsuko Hikima, Michael J. Jackson, Peter Jenner, K. Ray Chaudhuri, Helen M. Cox, Mahmoud M. Iravani, Sarah Rose

**Affiliations:** 1 Neurodegenerative Diseases Research Group, Institute of Cancer and Pharmaceutical Sciences, Faculty of Life Sciences and Medicine, King’s College London, London, SE1 1UL UK; 2 Wolfson Centre for Age-Related Diseases, IoPPN, King’s College London, Guy’s Campus, London, SE1 1UL UK; 3 Department of Clinical and Pharmaceutical Sciences, University of Hertfordshire, Hatfield, AL10 9AB UK; 4 Institute of Psychiatry, Psychology & Neuroscience, King’s College London, London, SE5 8AF UK; 5 Parkinson Foundation International Centre of Excellence, King’s College Hospital, Denmark Hill, London, SE5 9RS UK

**Keywords:** Ion channels in the nervous system, Neurophysiology

## Abstract

The pathological changes underlying gastrointestinal (GI) dysfunction in Parkinson’s disease (PD) are poorly understood and the symptoms remain inadequately treated. In this study we compared the functional and neurochemical changes in the enteric nervous system in the colon of adult, L-DOPA-responsive, 1-methyl-4-phenyl-1,2,3,6-tetrahydropyridine (MPTP)-treated common marmoset, with naïve controls. Measurement of mucosal vectorial ion transport, spontaneous longitudinal smooth muscle activity and immunohistochemical assessment of intrinsic innervation were each performed in discrete colonic regions of naïve and MPTP-treated marmosets. The basal short circuit current (*I*_sc_) was lower in MPTP-treated colonic mucosa while mucosal resistance was unchanged. There was no difference in basal cholinergic tone, however, there was an increased excitatory cholinergic response in MPTP-treated tissues when NOS was blocked with L-Nω-nitroarginine. The amplitude and frequency of spontaneous contractions in longitudinal smooth muscle as well as carbachol-evoked post-junctional contractile responses were unaltered, despite a decrease in choline acetyltransferase and an increase in the vasoactive intestinal polypeptide neuron numbers per ganglion in the proximal colon. There was a low-level inflammation in the proximal but not the distal colon accompanied by a change in α-synuclein immunoreactivity. This study suggests that MPTP treatment produces long-term alterations in colonic mucosal function associated with amplified muscarinic mucosal activity but decreased cholinergic innervation in myenteric plexi and increased nitrergic enteric neurotransmission. This suggests that long-term changes in either central or peripheral dopaminergic neurotransmission may lead to adaptive changes in colonic function resulting in alterations in ion transport across mucosal epithelia that may result in GI dysfunction in PD.

## Introduction

Parkinson’s disease (PD) is primarily considered to be a movement disorder resulting from the loss of dopaminergic innervation of the basal ganglia^1^. However, numerous non-motor symptoms are also present, and these may start before, at the same time or following the onset of motor symptoms^2^. Gastrointestinal (GI) dysfunction including dysphagia, gastroparesis, constipation and a decreased frequency of bowel movements all occur in PD, but it is the early appearance of a range of non-motor symptoms including constipation that precedes the onset of motor signs^3–6^. Constipation remains a significant problem throughout the course of PD and adversely affects the quality of life of the patient population^7^.

Gut function is finely controlled by innervation from both the peripheral and central nervous systems^8^. The intrinsic enteric nervous system (ENS) consists of the myenteric plexus, which predominantly controls longitudinal and circular muscle function, and the submucosal plexus, which controls mucosal function and the smooth muscle activity of the muscularis mucosae^8^. Although the transmission in these areas is controlled by a wide range of neurotransmitters, the main excitatory transmitter is acetylcholine and the main inhibitory transmitters are vasoactive intestinal peptide (VIP) and nitric oxide^8^. Peristaltic movement in the GI tract and regulation of fluid across the epithelial lining is controlled by the ENS, and this is crucial for effective peristalsis. While dopamine (DA) replacement therapy relieves the motor symptoms of PD, constipation is largely unaffected or worsened^9^. This suggests that the basis of GI dysfunction in PD is locally mediated and largely non-dopaminergic in nature.

GI dysfunction in PD may relate to early pathology in the dorsal motor nucleus of the vagus (DMV) and the loss of vagal tone^10^. Loss of nigral dopaminergic input to the DMV results in alterations in the peristaltic activity of the lower GI tract^11^, and in rats vagotomy prevented reduction of gut motility following paraquat administration^12^.

The enteric neurons within the myenteric plexus modulate motility, while neurons present in the inner submucous plexus modulate mucosal ion and fluid transport^8^. Both ganglionated networks contain sensory, as well as excitatory (e.g. cholinergic) and inhibitory (e.g. nitrergic and VIPergic) neurons which are involved in the reflexes that underpin propulsive activity in the small intestine and colon. More speculatively, based on early α-synuclein accumulation in the gut in PD^13^ and the ability of focal toxin treatment in the gut and inflammatory change to cause cell loss in the DMV^14^, there are suggestions that the disease process in PD may originate in the GI tract and progress retrogradely via the vagus into the brain^15^. However, it is not known whether the onset of gut dysfunction in PD that leads to constipation is initiated as a consequence of DA loss in the basal ganglia or whether it is due to functional and neurochemical adaptive changes at the level of the gut and/or ongoing inflammation.

In vivo functional alterations of colonic motility have been evaluated previously in rodent models of central dopaminergic loss. In the MPTP-, rotenone- and paraquat-treated mice and rats a delay of colonic transit and constipation have been identified^11,14,15^. Increases in contractile activity and impaired relaxation of the proximal colon have also been reported^10,16,17^. In 6-OHDA-treated rats, a significant decrease in the frequency of stools produced and a delay in colonic transit were observed^18,19^. Reports of alterations in neurochemical markers have been more variable. In the rotenone mouse model no changes were reported in nitrergic, VIP-ergic or dopaminergic innervation^20^. However, in unilaterally lesioned 6-OHDA rats there was an increase in VIP-ergic and dopaminergic innervation and a decrease in nitrergic neurons^19,21,22^. A study by Tian et al.^23^ described a decrease in colonic tyrosine hydroxylase (TH) expression in MPTP-treated mice, whilst in the 6-OHDA rat an increase in the levels of TH and the dopamine transporter (DAT) was found^24^. Reports on changes in the colonic excitatory cholinergic system have also produced conflicting results. In the rotenone model, ChAT immunoreactivity was reduced but two 6-OHDA rat studies reported no changes in ChAT, whereas one suggested a decrease in ChAT immunoreactivity in the distal colon^18,19,22,25^.

It is clear that there are changes to bowel function in experimental models of PD in rodents but with conflicting views on the neurochemical cause or consequence. In addition, there has been little work carried out in the model closest to man. While anecdotally we have some evidence of marked bowel dysfunction following very early stages of MPTP treatment, it has not always been possible to systematically study gut dysfunction throughout the life of these primates because the main focus of investigations in MPTP-treated common marmosets has been movement disorders. Previously, Chaumette et al.^26^ identified a reduction in TH-, and an increase in nNOS-immunoreactive (-ir) neurons in the MPTP-treated macaques, and we have shown a dysregulation of nitric oxide signal coupling in the ileum of MPTP-treated common marmosets, accompanied by a selective reduction in enteric cholinergic neurons^26,27^. However, there have been no studies of the changes in mucosal ion transport in the colon or the contractility of colonic tissue in isolated preparations, or accompanying changes in neuronal populations. As a consequence, this study investigated the neuro-epithelial signalling in colonic mucosa and the contractile responses of longitudinal smooth muscle in the MPTP-treated marmoset. In addition, we investigated changes in colonic neurotransmitters in an attempt to clarify the current discrepancies that exist.

## Results

**Fig. 1:**
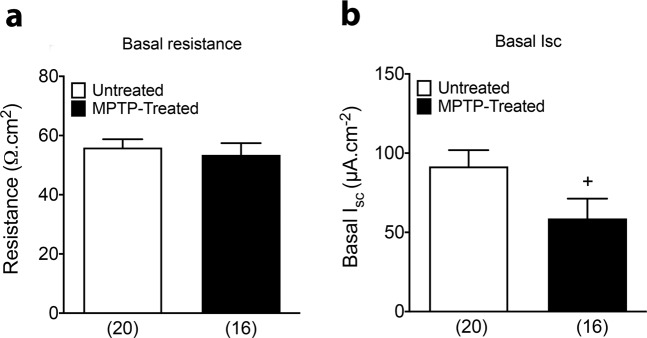
Basal resistance and I_SC_ levels in the transverse colon from untreated and MPTP-treated common marmosets. **a** Basal resistance; **b** short circuit current (I_SC_). Values are mean ± SEM from 4 or 5 specimens, as shown in parenthesis (*n* = 4 MPTP and *n* = 5 control). ^+^*P* ≤ 0.05 compared to untreated animals (Student’s *t*-test).

**Fig. 2:**
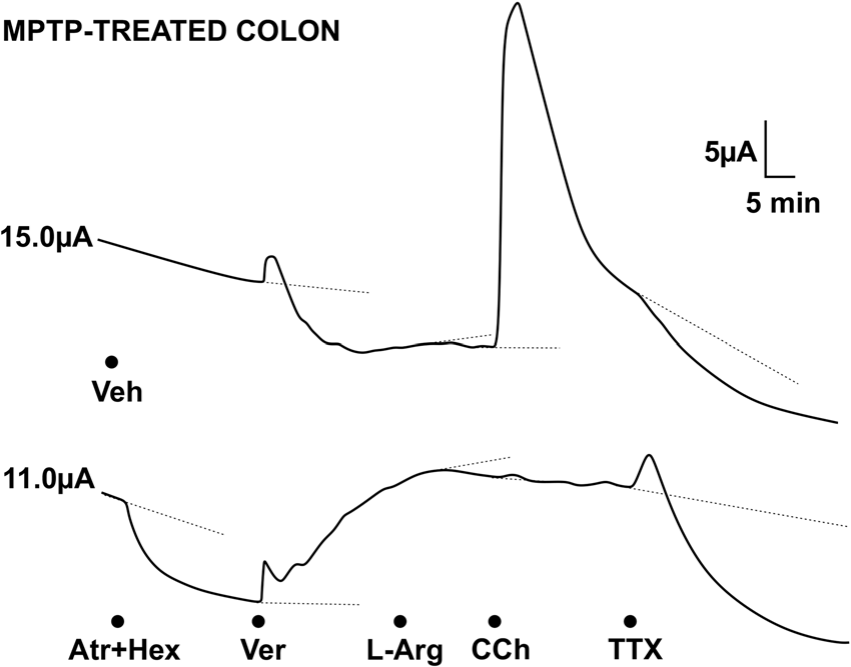
Typical traces from I_SC_ measurements in the transverse colon mucosa of an MPTP-treated marmoset. The upper trace is a vehicle (Veh) control for the lower trace where cholinergic antagonists, atropine and hexamethonium (Atr + Hex) were added first. Subsequent changes in I_SC_ in both preparations are evident after addition of veratridine (Ver), then 20 min later L-Arg, followed by CCh (note this response is blocked by the cholinergic antagonists) and finally, TTX was added to both preparations. Values to the left of each trace are the basal I_SC_ levels. Dotted lines show the extrapolated I_SC_ levels from which drug-induced maximum or minimum changes were measured between 5 and 20 min, as appropriate.

**Fig. 3:**
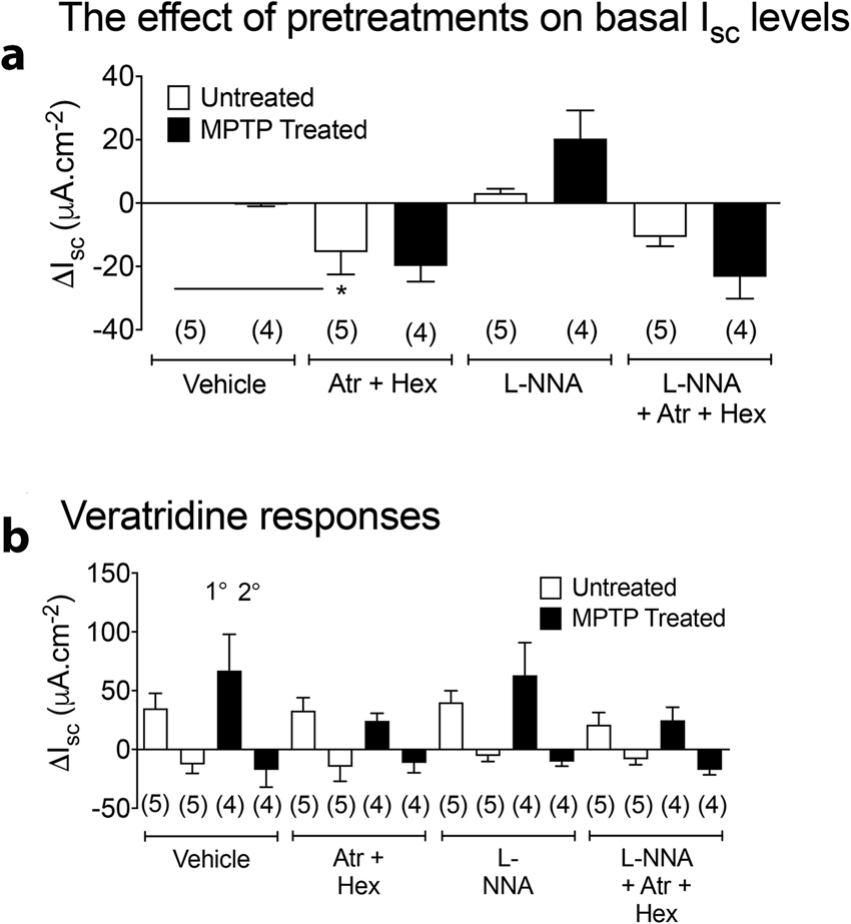
The effect of cholinergic and nitrergic antagonists. The effect of cholinergic and nitrergic antagonists alone (**a**) and following subsequent veratridine-induced mucosal responses (**b**) on the change in I_SC_ levels in transverse colon from untreated or MPTP-treated marmosets. Tissues were treated with either atropine plus hexamethonium (Atr+Hex) alone, L-NNA alone or Atr + Hex + L-NNA (**a**, **b**). Subsequent veratridine-induced changes in I_SC_ are divided into an initial 1°, and a slower 2° component after each pre-treatment (**b**). Values are change in I_SC_ (mean±SEM). Replicates are shown in parentheses. ^*^P ≤ 0.05 compared to vehicle treatment (ANOVA and Dunnett’s test).

There was no difference in the basal epithelial resistance values of colonic mucosa from untreated and MPTP-treated marmosets (Fig. 1a). By contrast, basal *I*_sc_ levels (which is derived from epithelial electrogenic ion transport) were significantly lower in mucosa from MPTP-treated animals compared with naïve controls (Fig. 1b). In order to investigate whether enteric neuro-epithelial signalling was altered we utilised the neuronal depolariser, veratridine that has been used previously to characterise neuro-epithelial mechanisms in mouse colon mucosa^28^ in the absence and presence of specific antagonists. Pre-treatment with cholinergic antagonists (atropine and hexamethonium; Atr + Hex) reduced basal *I*_sc_ levels compared with vehicle control (Fig. 2) but there was no significant difference in this tonic cholinergic activity between untreated and MPTP-treated groups (Fig. 3a). There were also no significant differences in *I*_sc_ responses to the nitric oxide synthase (NOS) blocker Nω-Nitro-L-arginine (L-NNA) alone, although the increase in *I*_sc_ to L-NNA tended to be larger in tissue from MPTP-treated animals, or in combination with cholinergic antagonists (Fig. 3a) indicating that the nitrergic as well as cholinergic tones within the submucosal innervation of colonic mucosa are not significantly altered. Depolarisation of the colonic submucosal innervation by veratridine caused initial rapid elevations in *I*_sc_ (the primary response; 1°) followed by changes that more often became reductions in *I*_sc_ (the secondary response; 2°) and these respective maxima and minima are presented separately in Fig. 3b. These complex neurogenic *I*_sc_ responses were similar in untreated and MPTP-treated mucosae and were not significantly altered by cholinergic or nitrergic blockade (or both) compared with vehicle controls (Fig. 3b). Tetrodotoxin (TTX) reversed the remaining veratridine-elevated *I*_sc_, confirming the neurogenic character of these biphasic veratridine responses (Fig. 2; pooled data not shown).

**Fig. 4:**
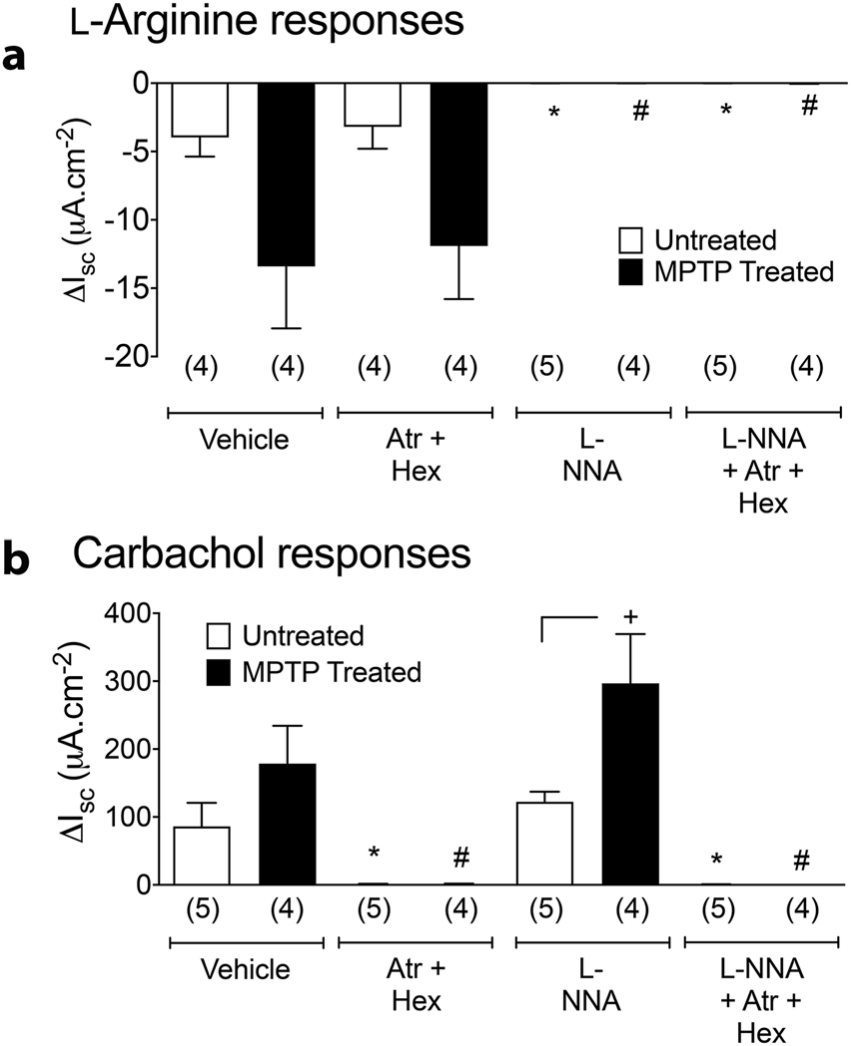
Changes in the short cuircuit current (I_SC_) in the presence of L-arginine or carbachol in transverse colon mucosa from naïve or MPTP-treated animals. **a** L-Arginine (200 µM) and **b** carbachol (10 µM). Values are mean ± SEM from the numbers of animals shown in parentheses. (**a**, **b**) ^*^P ≤ 0.05 compared to vehicle in naïve animals; ^#^*P* ≤ 0.05 compared to vehicle in MPTP-treated animals (ANOVA with Dunnett’s test).

The nitric oxide donor/precursor, L-Arg, reduced *I*_sc_ levels (after veratridine) and these responses were increased in MPTP-treated mucosa (with or without cholinergic blockade), although this was not statistically significant (Fig. 4a). Internal controls show that L-Arg responses were abolished predictably by L-NNA pre-teatment, but not by the cholinergic blockers (Fig. 4a). In contrast, the muscarinic agonist carbachol (CCh) elevated the *I*_sc_ levels and this activity was increased significantly in MPTP-treated tissues when inhibitory nitrergic mechanisms were blocked with L-NNA (Fig. 4b). As expected CCh responses were abolished by the cholinergic antagonists, atropine and hexamethonium and were not sensitive to L-NNA (Fig. 4b). In summary, significantly increased post-junctional muscarinic activity was evident in mucosae where nitrergic inhibition was reduced in the transverse colon from MPTP-treated animals. A degree of enhanced nitrergic signalling was also observed (but this was not statistically significant) in tissue from MPTP-treated colon.

### Functional changes in longitudinal muscle contraction

**Fig. 5:**
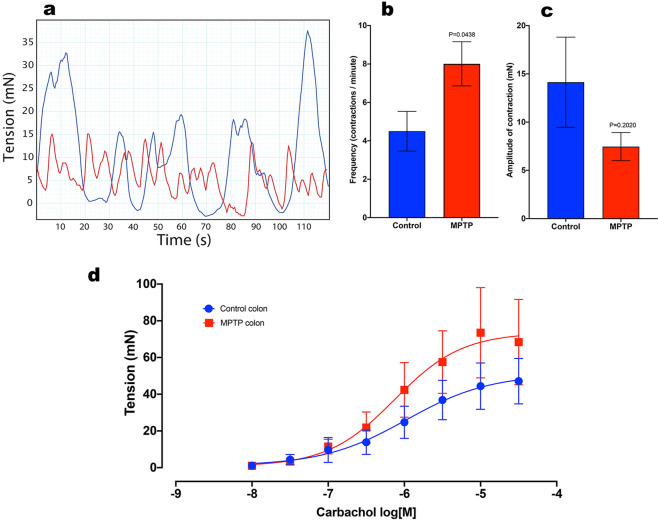
Frequency and the amplitude of spontaneous contractions in isolated distal colon preparation. **a** Two representative traces showing spontaneous contractile response of colon preparations obtained from control (blue trace) and MPTP-treated (red trace) animals. **b** The frequency and **c** amplitude of spontaneous contraction in MPTP-treated (*n* = 6) and naïve (*n* = 7) animals; unpaired Student’s *t*-test. **d** The effect of carbachol on the change in tone of isolated distal colon in MPTP-treated (*n* = 6) and naïve (*n* = 8) animals); all values are mean ± SEM; Two-way repeated measure ANOVA.

In isolated colon preparations from normal and MPTP-treated animals there were differences in the pattern of spontaneous contractions (Fig. 5a). Frequency of spontaneous contraction was significantly increased in tissues from MPTP-treated animals (Fig. 5b), whereas the amplitude of the contraction was slightly reduced although this was not significantly different (Fig. 5c).

Administration of CCh at concentrations ranging from 0.01 to 30 µM led to concentration-dependent contractions of the descending colon preparations obtained from normal and MPTP-treated animals (F (7,88) = 7.68, *P* < 0.001, two-way ANOVA; Fig. 5d). However, there were no significant differences between the responses obtained from the normal or MPTP-treated animals (F (1,88); *P* = 0.466, *P* > 0.05, two-way ANOVA; *n* = 7).

### Quantitative analysis of serotonergic and dopaminergic neurons

Analysis of immunohistochemical staining in the colon for the pan-neuronal marker HuC/HuD showed no overall difference in the total number of neurons in the myenteric plexus in proximal or distal colon in the MPTP-treated animals compared to naïve controls (Table 1). Analysis of double immunohistochemistry revealed that 1.0 % (proximal) and 0.6% (distal) of the myenteric neuronal population in the colon of common marmosets were TH-ir (Table 1). By contrast, approximately 21% of the myenteric neuronal population in the colon were serotonergic and approximately 32% of the neuronal population were immunoreactive for substance P (Table 1). Prior treatment with MPTP produced no long-term change in the number of dopaminergic or serotonergic or substance P immunoreactive neurons in either the proximal or distal colonic myenteric plexus.

**Table 1:** The number of 5-HT-, TH- and substance P-ir cells in an area of 1 mm^2^ relation to the number of Huc/HuD-ir neurons. Each value is mean ± SEM. There were no significant differences between the number of HuC/HuD-ir cells or other markers in the proximal or distal colon in either control or tissues obtained from MPTP-treated animals.

	Number of immunoreactive neurones/mm^2^					
Markers/regions	Huc/HuD-ir	5-HT-ir	Huc/HuD-ir	Tyrosine hydroxylase-ir	Huc/HuD-ir	Substance P-ir
Proximal colon - Control	1445 ± 64.4, *n* = 7	302 ± 15.6, *n* = 7	1251 ± 44.6, *n* = 6	12 ± 6.4, *n* = 6	1495 ± 74.8, *n* = 5	479 ± 45.6, *n* = 5
Proximal colon - MPTP	1610 ± 102.4, *n* = 6	339 ± 24.6, *n* = 6	1416 ± 64.5, *n* = 6	12 ± 3.9, *n* = 6	1595 ± 75.8, *n* = 4	529 ± 99.3, *n* = 4
Distal colon - Control	1516 ± 59.5, *n* = 8	313 ± 13.0, *n* = 8	1580 ± 125.9, *n* = 8	9.5 ± 3.4, *n* = 8	1541 ± 37.5, *n* = 7	538 ± 45.1, *n* = 7
Distal colon - MPTP	1484 ± 68.1, *n* = 5	353 ± 22.8, *n* = 5	1546 ± 58.9, *n* = 6	8.4 ± 4.6, *n* = 6	1699 ± 95.4, *n* = 4	550 ± 24.8, *n* = 4

### Quantitative analysis of inhibitory myenteric neuronal populations

**Fig. 6:**
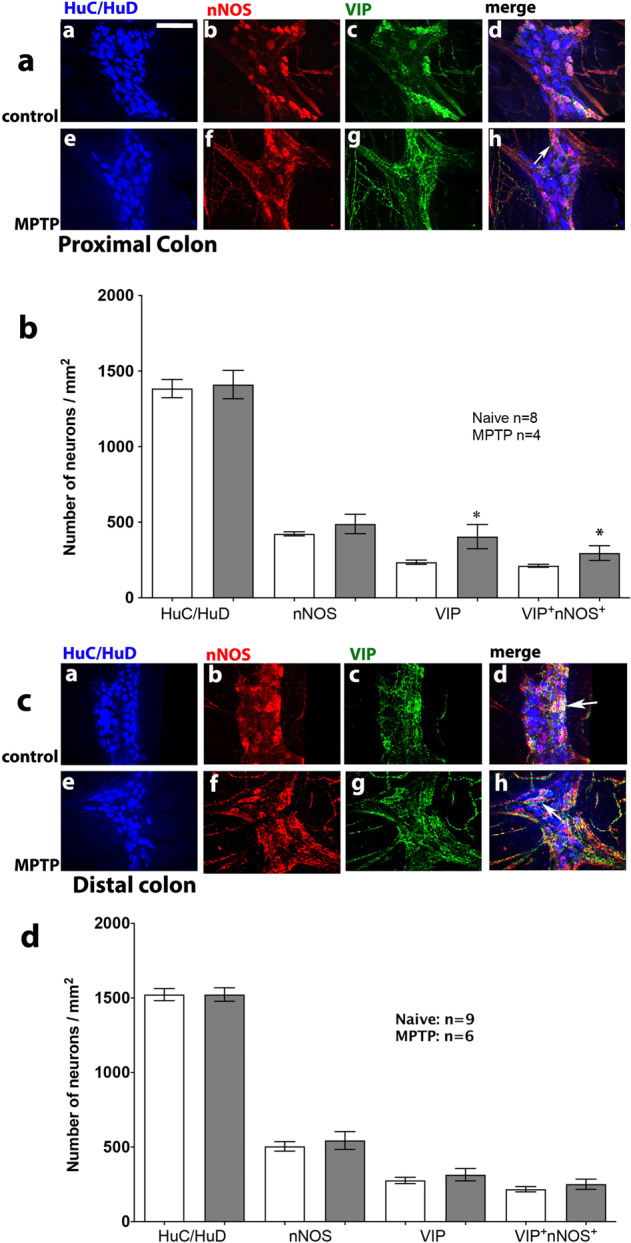
Effect of MPTP treatment on the number of HuC/D-, nNOS- and VIP-ir, in the proximal and distal colon of naïve control and MPTP-treated common marmosets. Inset panels (**a**, **e**) show HuC/D-ir; (**b**, **f**) show nNOS-ir and (**c**, **g**) show VIP-ir in the proximal (**a**) and distal (**c**) colon of naïve control and MPTP-treated common marmosets. Panels (**d**, **h**) show triple immunofluorescence for HuC/HuD (blue), nNOS (red) and VIP (green) in the myenteric plexus from proximal (main panel **a**) and distal (main panel **c**) colon of naϊve and MPTP-treated animals. Quantitative analysis of nNOS- and VIP-ir neurons in MPTP-treated marmosets compared to naϊve controls in the proximal and distal colon is shown in the main panels (**b**) and (**d**), respectively. The number of immunoreactive neurons are expressed as mean ± SEM. *P < 0.05 compared to naïve control (Students *t*-test). White arrows indicate co-localisation of nNOS and VIP. The scale bar in panel (**aa**) indicates 100 µm.

The number of VIP-ir neurons was significantly increased in the proximal but not in the distal colon in the MPTP-treated marmosets (Fig. 6a–d). Whilst analysis of the number of neurons expressing both VIP-ir and nNOS-ir showed no quantitative changes in either segment of the colon, the number of neurons expressing VIP-ir or colocalised VIP and nNOS-ir in MPTP-treated marmosets was significantly increased in the proximal colon (Fig. 6a–d).

**Fig. 7:**
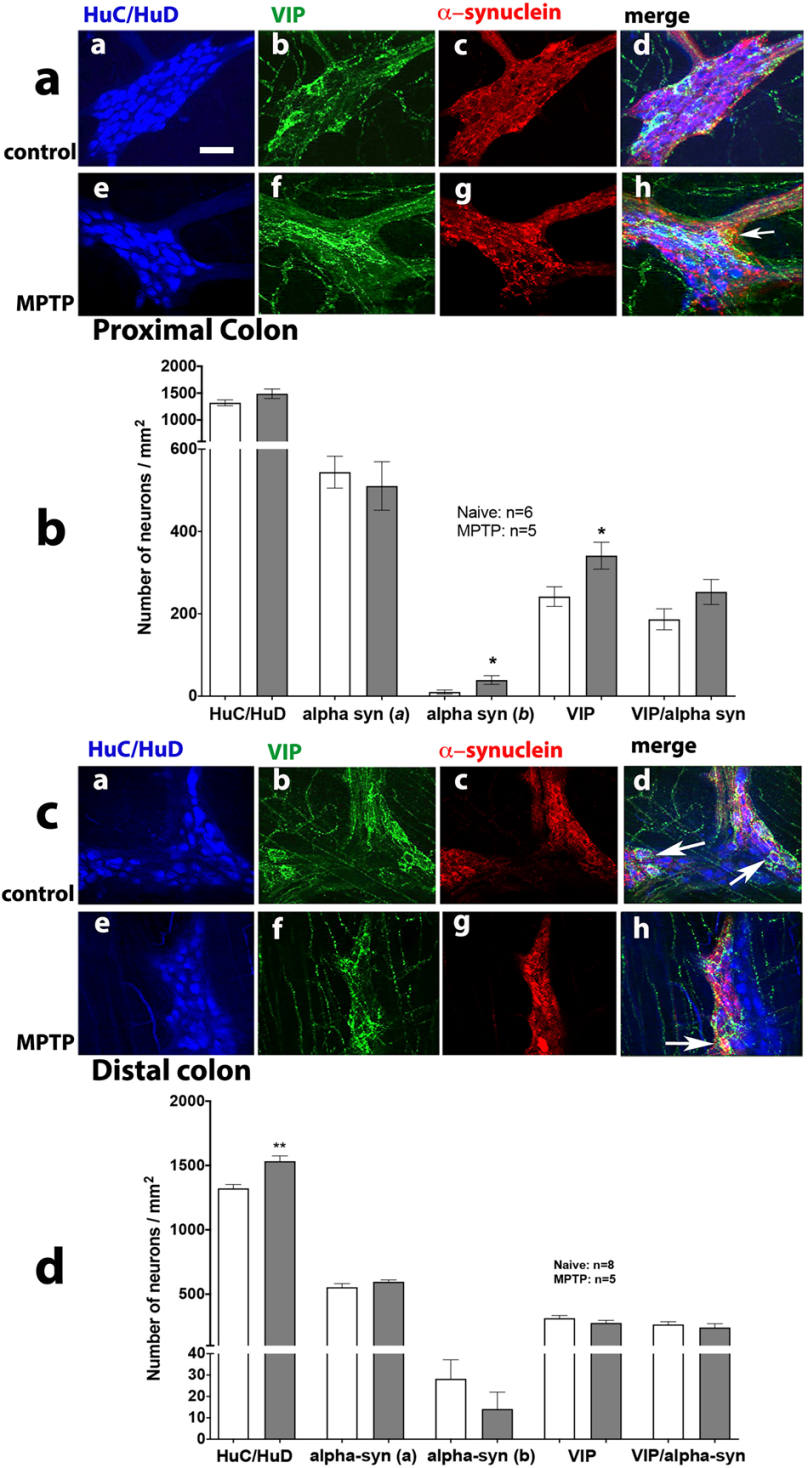
Effect of MPTP treatment on the number of HuC/D-, VIP- and α-synuclein-ir in the proximal and distal colon of naïve control and MPTP-treated common marmosets. Inset panels (**a**, **e**) show HuC/D-ir; (**b**, **f**) show VIP-ir and (**c**, **g**) show α-synuclein-ir in the proximal (**a**) and distal (**c**) colon of naïve control and MPTP-treated common marmosets. Panels (**d, h**) show triple immunofluorescence for HuC/HuD- (blue), VIP- (green) and α-synuclein-ir (red) in the myenteric plexus from proximal (main panel **a**) and distal (main panel **c**) colon of naϊve and MPTP-treated animals. Quantitative analysis of VIP-ir and α-synuclein-ir (**a**: soma; **b**: extracellular surrounds) neurons in MPTP-treated marmosets compared to naϊve controls in the proximal and distal colon is shown in the main panels (**b**) and (**d**), respectively. The number of immunoreactive neurons are expressed as mean ± SEM. *P < 0.05 compared to naïve control (Students *t*-test). White arrows indicate co-localisation of VIP- and α-synuclein-ir. The scale bar in panel (**aa**) indicates 100 µm.

We investigated the distribution of α-synuclein immunoreactivity in the proximal and distal colon (Fig. 7). Around 40% of the HuC/HuD-ir neurons in the myenteric plexus were associated with α-synuclein-ir, the majority of which was found localised perineuronally (Fig. 7c). There was no change in the total number of neurons containing α-synuclein in the MPTP-treated animals in the distal colon, however, there was a significant increase in the number of α-synuclein-ir soma in the proximal colon (Fig. 7b, d). In addition, in the proximal but not distal colon the distribution of α-synuclein also changed from the predominantly perineuronal to a more diffuse cytoplasmic distribution (Fig. 7a, c).

### Quantitative analysis of excitatory cholinergic pathways

**Fig. 8:**
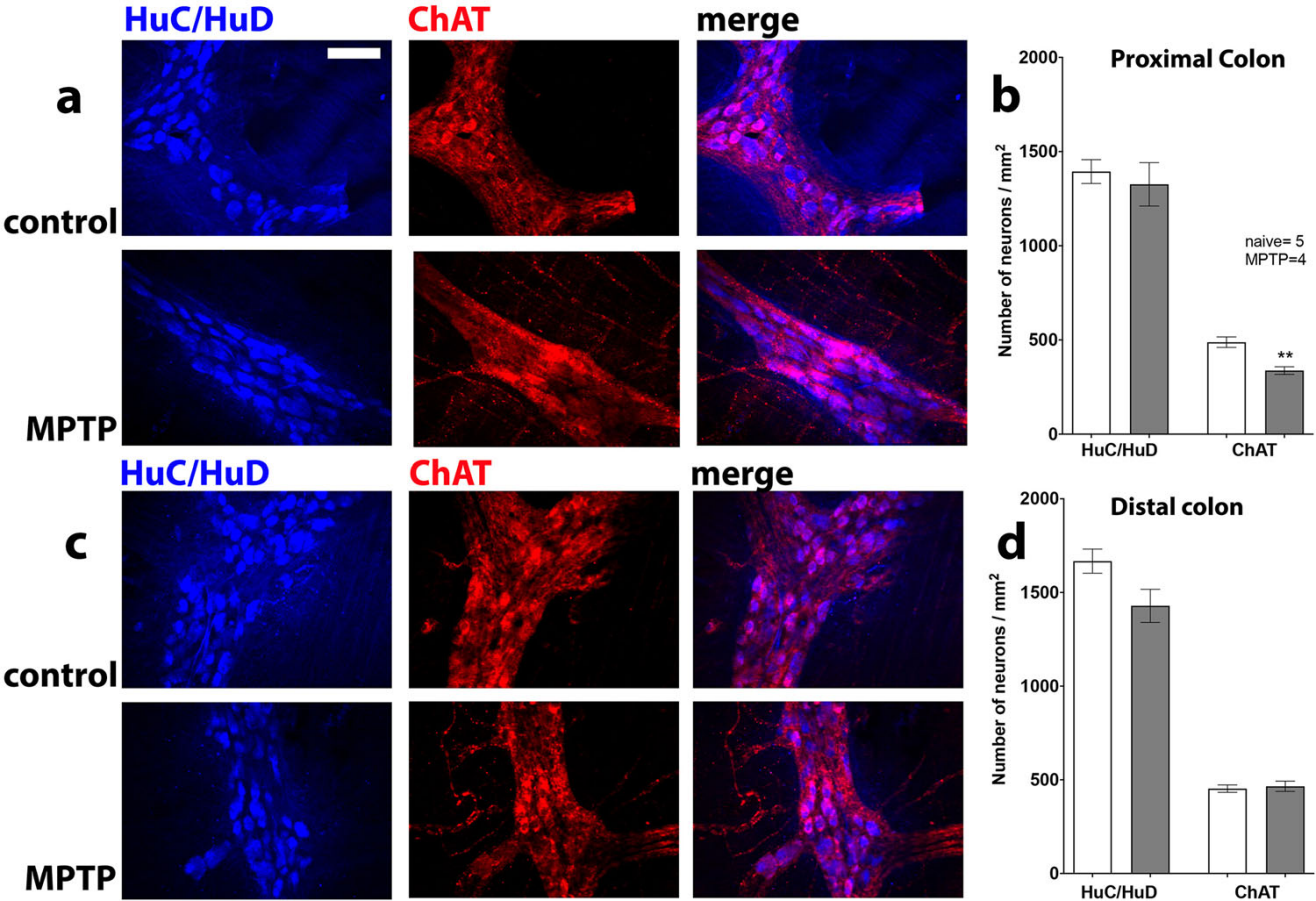
Effect of MPTP treatment on ChAT / HuC/HuD-ir neurons in the proximal and distal colon. Panels (**a**, **b**) show proximal and (**c**, **d**) show distal colon. Quantitative analysis of HuC/HuD- and ChAT-ir neurons in MPTP-treated marmosets compared to naϊve controls in the proximal and distal colon is shown in panels (**b**) and (**d**), respectively. The number of immunoreactive neurons are expressed as mean ± SEM. **P < 0.01 compared to naïve control (Students *t*-test). The scale bar in panel (**a**) indicates 100 µm.

The assessment of the number of ChAT containing neurons was made by double immunofluorescence labelling with HuC/HuD (Fig. 8). There was a significant decrease in the number of ChAT-ir neurons in the proximal but not distal colon of MPTP-treated animals, whilst the total number of myenteric neurons was unchanged (Fig. 8a–d).

### Quantitative analysis of enteric glial cells

**Fig. 9:**
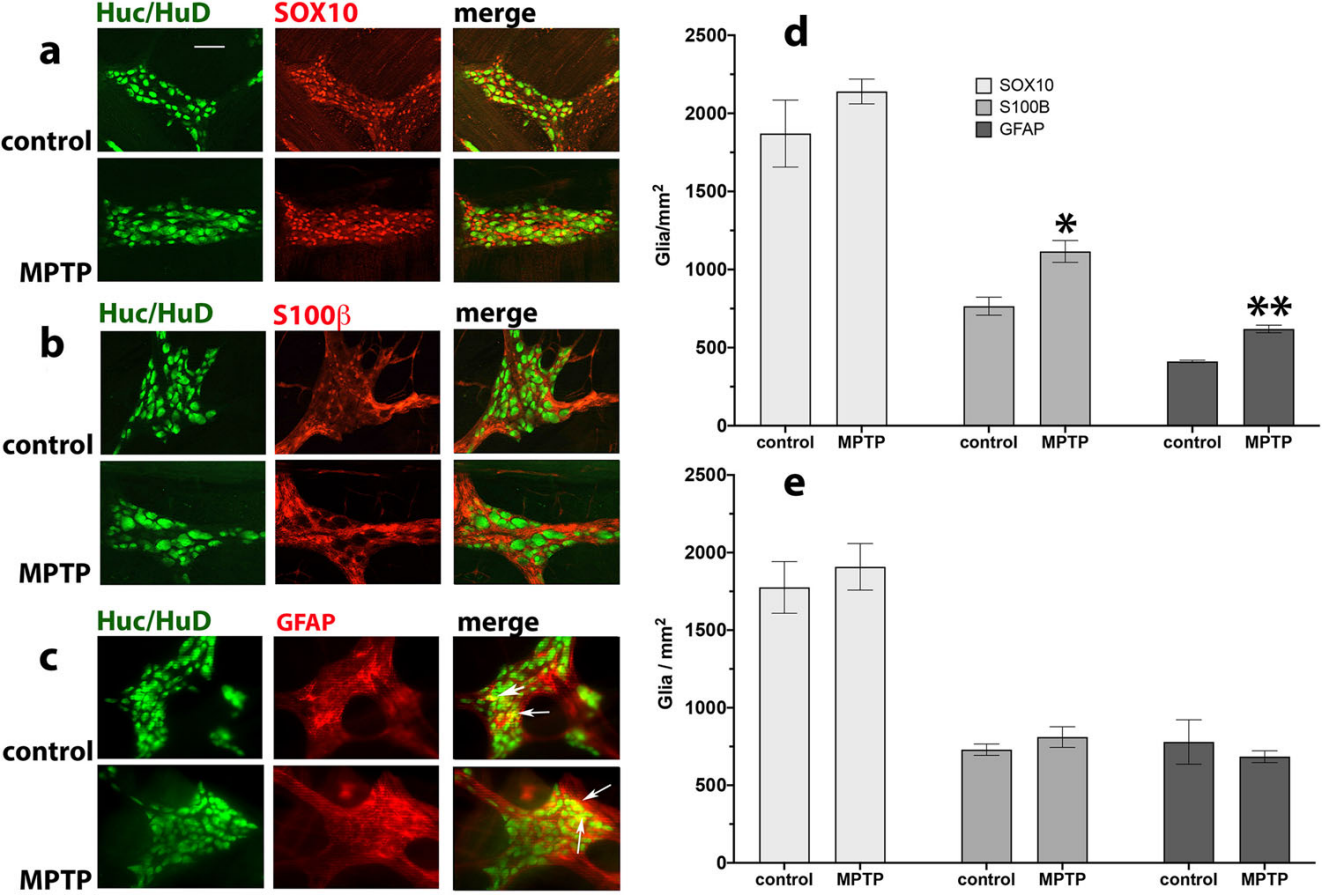
Effect of MPTP treatment on SOX-10-, S100β- and GFAP-ir glial cells in the myenteric plexus of the proximal colon of naïve control and MPTP-treated common marmosets. Panel (**a**) shows SOX-10-ir, (**b**) S100β-ir and (**c**) GFAP-ir. With the exception of GFAP-ir (**c**) there was no signal overlap between the immunoreactivity of glial and neuronal cells. The white arrows indicate colocalisation of GFAP-ir and Huc/HuD-ir cells. The arrows indicate glial/neuronal overlap. Quantitative analysis of glial cells in MPTP-treated marmosets compared to naϊve controls in the proximal and distal colon is shown in panels (**d**) and (**e**), respectively. The number of immunoreactive neurons are expressed as mean ± SEM. Scale bar in panel (**a**) indicates 100 µm. *P = 0.02; **P = 0.0016 two-way ANOVA, Sidak multiple comparison test.

There was an extensive presence of S100β-, GFAP- and SOX-10-ir inflammatory markers in both proximal and distal colon that rarely colocalised with HuC/HuD-ir cells. The number of S100β-, GFAP- and SOX-10-ir cells was determined in the myenteric plexus of the proximal and distal colon on MPTP-treated and naïve control marmosets. Whilst compared to the neuronal numbers the number of SOX-10-ir glia was significantly greater than that of S100β- or GFAP-ir cells in both the proximal and distal colon, MPTP treatment did not result in a significant change in their number or distribution. However, there was a significant increase in the number of S100β-ir and GFAP-ir in proximal but not distal colon following MPTP treatment. Moreover, there was some evidence of GFAP-ir overlap with HuC/HuD-ir in the distal colon, but overall there was no glial overlap between HuC/HuD-ir cells suggesting the distribution of SOX-10, S100β-ir and GFAP-ir was glial (Fig. 9).

## Discussion

Reduced gut motility and constipation are common problems associated with changes on colon function in PD. This has been reproduced in both rodent and primate models of PD where the nigro-striatal pathway has been destroyed^6,18,22,26^. However, there have been no studies connecting the histological changes with functional alterations at cellular and physiological level. Specifically, no one to our knowledge has investigated connections between these changes in the MPTP-treated primate model of PD. We now show functional, biochemical and physiological changes in common marmosets in the colon over 1 year after MPTP treatment suggesting long-term local adaptive change and possible alteration in central control of gut function.

The majority of neurons in the myenteric plexus of naïve animals were serotonergic (21%), cholinergic (35%), nitrergic (40%), substance P-ergic (32%) or VIP-ergic (17%), with the expression of neurons containing TH-ir being sparse (<1%). Previous studies in untreated macaques showed a similar profile, although the proportion of VIP-ergic neurons was much lower than in the marmoset^26^. Previously Natale et al.^29^ reported a reduction in murine enteric dopaminergic neurons 1 week after MPTP treatment in the duodenum but not in the colon. Whilst we found no change in the number of TH-ir neurons in the colon, we have previously reported generalised reduction in the intensity of TH-ir staining in the ileum but not the actual cell numbers^27^ suggesting that these neurons may not be as susceptible to the toxic effects of MPTP as the dopaminergic neurons of the substantia nigra were, or were better able to repair over time. However, because TH is a common enzyme in the biosynthesis of both dopamine and noradrenaline much of the sparse TH immunoreactivity in the colon is likely to be associated with the extrinsic sympathetic innervation^30^, which may explain why the selective dopaminergic toxin MPTP did not alter the TH-immunoreactivity in the myenteric plexus of the common marmosets.

The number of cholinergic neurons in the myenteric plexus was reduced by 27% in the MPTP-treated marmosets compared to naïve controls, whereas the number of VIP-ergic neurons almost doubled. Interestingly, there was no change in the number of nNOS-ir cells. These findings are contrary to the changes reported in MPTP-treated macaques where the total neuronal population (HuC/HuD-ir) and the number of nitrergic neurons increased, but there were no changes in cholinergic or VIP-ergic cell number^26^. There is considerable diversity in the changes in neuronal populations in the different animal models of PD which may reflect the species, toxin, time and recovery differences. In the MPTP-treated mouse there was no change in nNOS-ir or ChAT-ir neurons 10 days after MPTP treatment^17^, and there was a functional recovery of the reduction of dopamine levels in the ileum over time^31^. However, recently Sampath et al.^32^ reported reduced nNOS expression on all parts of the colon 7 days after MPTP treatment, with a reduction in dimer formation suggesting reduced nNOS activity. In unilateral 6-OHDA-lesioned rats the number of ChAT-ir neurons were reported as decreased or unchanged, whereas there was a reduction in nitrergic and increase in VIP-ergic neurons^21,22,33^, partially agreeing with the present study. All the models used showed a reduction in nigral dopamine cell bodies, however, this was greatest in the 6-OHDA-lesioned rat (>90% loss) and the present study (>70% loss; data not shown). Whilst it is not possible to make a definitive conclusion, the data in the 6-OHDA-lesioned rat and the MPTP-treated marmoset may suggest a central component to these changes, as no toxin was administered systemically in the rodent model, so there would be no direct effect on the gut tissue. In addition, the time after MPTP treatment in the marmosets in the present study would allow acute toxic damage to the gut to be reversed as eluded to in the MPTP mouse where recovery of the neurochemical changes was seen over time^31^. In the current study we observed a significant but low-level inflammation exemplified by the lack of change in SOX-10 glial markers and an increase in GFAP- and S100β in the proximal but not in the distal colon. In our previous study^27^ we did observe a modest but significant increase in the number of SOX-10-ir cells in the ileum following MPTP treatment, but the lack of change in the proximal and distal colon in this study may suggest a regional pattern in the expression of inflammation.

The evidence of an inflammatory response in the proximal colon, albeit mild, is in accord with the reported increase in the expression of pro-inflammatory and glial marker in PD following colonoscopy of the ascending colon^34^, which roughly corresponds to proximal colon in this study, and in the colon of unilaterally 6-OHDA-lesioned rats^33^. However, the magnitude of difference observed between our study and those in the human PD and 6-OHDA-lesioned rats is not surprising as in PD the degeneration is progressive, and in rats the samples were obtained 8 weeks after lesioning^33^ while the neuronal cell loss in the MPTP-treated primate is acute and the samples were taken approximately 1.4 years following MPTP lesioning, thus allowing for some recovery from an inflammatory response. Indeed, repair is observed only up to 24 weeks where new neurons appear in the ENS, and only after 5-HT4 receptor stimulation^35^. Interestingly, Devos et al.^34^ also showed the presence of phosphorylated α-synuclein immunoreactivity in two PD subjects undergoing colonoscopy in the ascending colon. Naïve marmosets showed a mainly perinuclear distribution of α-synuclein in approximately 30% of the neuronal cells of the ENS, whereas over 1 year after MPTP treatment, the distribution of α-synuclein was more diffuse. We have previously shown that MPTP treatment impairs proteasomal enzyme activity in common marmosets which is intimately linked to the onset of synucleinopathies^36^ and more recently, Li et al.^37^ showed that 32–36 days following MPTP in cynomolgus monkeys, there was a significant increase in the level of total, phosphorylated and oligomeric α-synuclein. Lai et al.^31^ reported that two days following MPTP treatment in mice, there was an initial reduction in α-synuclein expression in the myenteric plexus of ileum but after 2–3 weeks there was a rebound increase with a corresponding increase in inflammation and pro-inflammatory cytokine production suggesting that MPTP did not acutely alter the level of α-synuclein expression but perhaps the increase in the level of α-synuclein was related to the onset of gut inflammation. In the present study, the expression and localisation of the α-synuclein following MPTP was very diffuse and its incidence and pattern of distribution which corresponded to the pattern of inflammation in the proximal colon suggest that the expression of α-synuclein is indeed linked with inflammatory changes.

To our knowledge, monitoring changes in mucosal ion transport to investigate colonic dysfunction at the cellular level in a model of PD have not been investigated previously. There was no effect of MPTP treatment on basal resistance suggesting that the colonic mucosa was intact and there was no significant deterioration of epithelial tight junctions. The reduced basal *I*_sc_ levels observed in colon of the MPTP-treated marmosets could be a consequence of reduced neurogenic tone, or reduced secretagogues from non-neuronal resident cells within the lamina propria. Further investigations will be required to identify the cellular mechanism(s) that contribute to this difference in basal activity. Depolarisation of submucosal innervation by veratridine stimulated complex net changes in *I*_sc_ that were not significantly altered following MPTP compared with normal colon mucosa. Veratridine-activated submucosal neurons, the majority of which innervate the mucosa and are either sensory, secretomotor or secretomotor-vasodilator neurons, each of which express and release different primary (as well as various secondary) neurotransmitters^8^ and may be differentially affected by MPTP treatment. Previous studies have shown that in mouse distal colon, the majority of the mucosal veratridine response is non-adrenergic and non-cholinergic (NANC), and not significantly altered by cholinergic blockade^28^. The same appears to be true in marmoset colon and the neuroeffectors are thus potentially neuropeptide(s) and/or NO, but further studies are required to identify the predominant submucosal neurotransmitters. Despite this complexity, it appears that prejunctional and presynaptic cholinergic mechanisms are not significantly altered in colonic tissue from MPTP-treated animals in spite of the 21% loss of cholinergic cell bodies in the proximal colon. However, muscarinic epithelial signalling (revealed by CCh addition) is significantly amplified (perhaps as a compensatory response to the reduced cholinergic transmission) and NO-mediated responses are increased in colonic mucosa from MPTP-treated animals. How this hyper-responsiveness in post-junctional muscarinic and nitrergic signalling translates into slowed motility and constipation has yet to be resolved, but increased nitrergic tone (revealed by L-NNA) might be expected to amplify mucosal anti-secretory effects (which we observed, Fig. 4a) and slower colonic motility. However, the immunohistochemical analysis of the myenteric plexus showed no difference in the number of nNOS immunoreactive cells per ganglia, but the number of ChAT-ir was significantly decreased. Although immunoreactivity was not assessed in the submucosal plexus, a similar decline in the cholinergic neurones in that region (in line with the decline in the cholinergic neurones of the myenteric plexus) might explain the alterations in the epithelial signalling. An earlier study suggested that neuropathological changes occurring in the GI tract following MPTP are more limited to the myenteric plexus than the submucosal plexus even though dopaminergic innervation was similarly reduced^26^. These changes may be the result of acute toxicity as no change in dopamine innervation was seen in the present study over 1.4 years after MPTP treatment. Pathological changes such as the presence of Lewy bodies do occur in both the submucosal and myenteric plexuses in PD^3,38–40^. Therefore, whether other neurochemical phenotypes such as VIP, acetylcholine and nitric oxide in the submucosal plexus are similarly patterned remains to be established but any neurochemical changes seen in the myenteric plexus are also likely to be reflected in the submucosal plexus. Three to five weeks following MPTP treatment, western blot analysis suggested α-synuclein levels were reportedly raised in the ileum of mice^31^ and colon of monkeys^37^. Whilst we did not measure levels of α-synuclein in the present study, we noted that there was a redistribution of the protein in the neurons of the proximal but not the distal colon such that α-synuclein was found to a greater extent in the whole neurons rather than surrounding the cell body.

The reduction in excitatory innervation and increase in the number of inhibitory (NOS-ir) neurons conflicts with the increased frequency and reduced amplitude of the contraction of the longitudinal muscle seen in colon from the MPTP-treated monkeys, although the observed changes to these spontaneous contractions may be explained by the ongoing inflammatory changes. Interestingly, the amplitude of electrically evoked contractions in rat colon is reduced, and there is a dysregulation of contraction ex vivo, following a 6-OHDA lesion, suggesting some local adaptive changes to centrally mediated effects^6,22^. The reduction of ChAT-ir neurons in the myenteric plexus of the MPTP marmosets in this study would predict that the contractile response of the longitudinal muscle of colon to exogenous CCh may be greater due to the phenomenon of denervation supersensitivity. However, there was no significant difference in the concentration-response curves to CCh suggesting that cholinergic control of longitudinal muscle was not altered significantly. Further investigations are needed to fully understand the role of changes in the ENS compared to the CNS in colonic activity.

In conclusion, the most striking finding of this study is that following MPTP treatment, there are specific adaptive physiological changes in the colon ion transport across the mucosal epithelia. Alterations in ion transport can have several important implications. These may include altered handling of Na^+^, Cl^−^, HCO_3_^−^ and K^+^ which collectively would influence electrolyte homoeostasis and water transport across the epithelia. Longer term such dysfunction might affect the balance and the proliferation of the GI microbiota (and their metabolites e.g. short chain fatty acids such as acetate, propionate, and butyrate, which are synthesised from dietary carbohydrates by colonic bacterial fermentation) which in turn alter mucosal function and slow colon motility^41^. It may be speculated that these changes could cause constipation, and may be a trigger for accumulation of misfolded α-synuclein in the gut^42^. The latter factor has been implicated in the pathogenesis of Parkinson’s disease via the gut–brain axis^43^.

## Methods

### Animals

Two groups of adult common marmosets (*Callithrix jacchus;* Harlan UK Ltd. Loughborough, LE12 9TE, UK; Manchester University and King’s College London) were used in this study. One group comprised of drug and toxin naïve controls (*n* = 8; 4 male and 4 female, 333–407 g, 373 ± 16 g) aged 3.0 ± 0.2 years. The second group (*n* = 7, 2 males and 5 female, 313–389 g, 359 ± 17 g), aged 3.8 ± 0.4 years had been previously treated with 1-methyl-4-phenyl-1,2,3,6-tetrahydropyridine (MPTP) 1.4 ± 0.4 years prior to this study. The animals were housed singly or in male (vasectomised)/female pairs (cage dimensions of height: 166 cm, width: 140 cm, depth: 90 cm), in a room maintained at temperature (24 ± 2 °C), 50% relative humidity and with a 12-h light/dark cycle. All animals were given 1 meal of mashed cereal and 1 meal of fresh fruit daily and had ad libitum access to food pellets and fresh water. Regulated procedures comply with the ARRIVE guidelines, were carried out in accordance with the UK Animals (Scientific Procedures) Act 1986, with approval of the King’s College London Animal Welfare and Ethical Review Board under project licence PPL 70/7146 and were compliant with the minimal standards as defined by the European Communities Council Directive (2010/63/EU). The animals’ environment was enriched by the installation of a viewing turret (height: 36 cm, width: 35 cm, depth: 50 cm) on top of the housing cages allowing the animals to climb above head height of research staff. Also provided were wooden ladders/perches, hammocks, swings, nesting boxes, multiple feeding platforms and saw dusted floors for forage feeding.

### MPTP treatment

Motor deficits were induced 1.4 ± 0.4 years prior to this study by the administration of 1-methyl-4-phenyl-1,2,3,6-tetrahydropyridine hydrochloride (MPTP; 2.0 mg/kg, sc; S.I.D. for up to 5 days, Sigma, UK)^44^. Once recovered from the acute effects of MPTP (approximately 3 months), all of the animals exhibited stable motor deficits including a marked reduction of locomotor activity, poor coordination of movement, abnormal and/or rigid posture and reduced alertness/head-checking movements. The MPTP-treated animals had previously been used for the behavioural assessment of response to antiparkinsonian drug treatment. All animals of this group were subjected to levodopa (8 mg/kg) plus benserazide (15 mg/kg), 3 animals in this group were also subjected to ropinirole (up to 1 mg/kg), an AMPA allosteric modulator and nitric oxide synthase inhibitors. All animals received a wash out period of at least 2 months prior to this study and there were no overt contractile differences between tissues obtained from these animals. Immunohistochemical studies showed >70% TH cell loss in the substantia nigra pars compacta compared to the naïve controls^27^.

### Perfusion of animals

The animals were euthanised using an overdose of pentobarbital sodium (60 mg/kg; Euthatal, Merial Animal Health Ltd.) between 7.30 and 8.30 a.m. Upon cessation of foot and corneal reflexes, the thoracic and abdominal cavities were opened. The animals were transcardially perfused with ice-cold oxygenated (95% O_2_ plus 5% CO_2_) Krebs-Henseleit (KH) solution (composition mM: NaCl, 118; KCI, 4.7; CaCl_2_, 2.5; MgSO_4_, 1.2; NaHCO_3_, 25; KH_2_PO_4_, 1.2; glucose, 11.1).

### Mucosal resistance and short-circuit current (*I*_sc_) measurement

Transverse colon (~5 cm taken midway between the caecum and rectum) was excised and placed in fresh KH buffer prior to microdissection. Mucosal preparations with intact submucosal innervation were prepared by removing the overlying smooth muscle and integral myenteric innervation. Four adjacent mucosal pieces were each mounted between two halves of the Ussing chambers (exposed area of 0.14 cm^2^) as described previously^41,45,46^. The tissues were bathed in KH (5 ml, both sides) at 37 °C, aerated with 95% O_2_/5%CO_2_ and voltage-clamped at 0 mV. After an equilibration period of 20–30 min the stable baseline short-circuit current (*I*_sc_) and transepithelial resistance were noted, and basolateral addition (unless otherwise stated) of drugs made, recording resultant changes in *I*_sc_ continuously. The mucosae were pre-treated for 20 min with either; (i) a mixture of atropine (1 µM) and hexamethonium (200 µM) to block cholinergic-mediated responses; or (ii) Nω-Nitro-L-arginine (L-NNA; 1 mM) to inhibit nitrergic responses; or (iii) all three inhibitors, with the fourth mucosal preparation being a vehicle control. Following these pre-treatments, the neuronal depolarising agent veratridine (30 µM) was added to establish the mucosal effects of enteric nerve stimulation and the altered *I*_sc_ was allowed to stabilise (20 min) before adding control agonists, namely L-arginine (L-Arg, 200 µM), and after a further 20 min CCh (10 µM), and finally in some experiments, tetrodotoxin (TTX, 100 nM) to block any remaining neural activity. The peak changes in *I*_sc_ to each of these agents were recorded at given time points. Data were pooled and changes in *I*_sc_ were expressed as mean ± 1 SEM (µA/cm^2^). Single comparisons utilised Student’s unpaired *t*-test, while multiple comparisons were performed using one-way ANOVA with Dunnett’s post-test, with a *P* value <0.05 being considered statistically significant.

### Colonic isometric contraction measurement

The descending colon was excised from the rectum to the start of the descending colon and placed in aerated KH. Three to four 1.5 cm lengths of the descending colon were cut transversely from the proximal end and suspended in a 15 ml organ baths with a resting tension of 1.0 g-force at 37 °C, as published previously^47^ with minor modifications. Following a 30 min equilibration period and initiation of spontaneous contractions, the colonic contractile activity was measured using an isometric transducer connected to LabChart data acquisition system with signal triggers to mark start of spontaneous and drug-induced contractile responses (AD Instruments Ltd., Oxford, UK).

Tissue viability was tested initially by administration of CCh (10 µM) to access the contractile responses. Only tissues that contracted >2 g-tension were included in the study. To assess whether prior MPTP treatment affected the receptor/effector coupling or whether there were changes at the level of smooth muscle, the isolated colonic preparations were contracted by cumulative addition of various concentrations of CCh (0.01–30 µM; Sigma Aldrich). The amplitude was estimated by adding together the tension of individual contractions occurring during a period of 10 min and dividing this sum by the number of contractions during this period. The rate of spontaneous activity (rate/min) was derived from dividing the total number of contractions during the 10 min observation time.

### Intestinal whole-mount preparation

Sections of the distal colon (1 cm from the rectum) were isolated and immersed for 15 min in phosphate buffered saline (PBS) pH 7.2 containing 1 µM nicardipine as a muscle relaxant (Sigma-Aldrich, UK). They were then cut along the mesenteric line and washed in PBS to remove luminal contents. Subsequently, they were stretched on a wax support with the mucosal layer downwards and fixed for 6/7 h in Zamboni’s fixative (2% paraformaldehyde containing 0.2% picric acid in 0.1 M PBS) at 4 °C. Following fixation, the tissues were washed in dimethylsulfoxide (DMSO) followed by three 10 min washes in PBS and then stored at 4 °C in PBS containing 0.1% sodium azide. Stretched specimens were processed as longitudinal muscle-myenteric plexus whole-mount preparations (LMMPs) by dissociating the different intestinal layers (i.e. mucosa, submucosa and circular muscle) as previously described^21^.

### Immunohistochemistry

To analyse enteric neurons and glial populations in myenteric plexus of the LMMPs, double staining [HuC/HuD-choline acetyl transferase (ChAT), HuC/HuD -5-HT, HuC/HuD-substance P (SP), HuC/HuD-tyrosine hydroxylase (TH), HuC/HuD-glial fibrillary acidic protein (GFAP), HuC/HuD-SOX-10 and HuC/HuD-S100β)] or triple labelling [HuC/HuD- vasoactive intestinal polypeptide (VIP)/neuronal nitric oxide synthase (nNOS), Hu/nNOS, HuC/HuD-α-synuclein/VIP)] immunofluorescence techniques were performed. Depending on the antibody characteristics either indirect immunohistochemistry or labelled streptavidin-biotin (LSAB) methods were used.

### The labelled streptavidin-biotin (LSAB) immunohistochemistry

After staining with primary antibodies, tissues were washed in PBS and then incubated with biotinylated secondary antibody, diluted in the blocking buffer, for 1 h at room temperature. Whole-mount preparations were then incubated overnight with a second primary antibody following the same procedure for time and temperature. After 24 h, specimens were washed in PBS and incubated in fluorescent dye streptavidin and suitable secondary antibody for 3 h, then washed and mounted as described above. All primary and secondary antibodies used are summarised in Table 2.

**Table 2:** Primary and secondary antibodies, their sources and dilutions.

1° Antibody	Host	Dilution	Source	2° Antibody	Dilution
Neuronal protein monoclonal HuC/D	Mouse	1:50	16A11: Molecular probes	Donkey anti-mouse Alexa fluor 350 IgG (H + L)	1:100
Vasoactive intestinal polypeptide polyclonal	Rabbit	1:100	Sc-20727; Santa Cruz Biotechnology	Horse FITC anti-rabbit IgG (H + L)	1:100
Neuronal nitric oxide synthase polyclonal	Rabbit	1:500	AB5380: Millipore	Goat FITC anti-rabbit IgG (H + L)	1:100
Tyrosine hydroxylase polyclonal	Rabbit	1:500	P40101; Pel-Freez Biologicals, USA	Biotinylated goat anti-rabbit-IgG (H + L)	1:200
Tyrosine hydroxylase polyclonal	Chicken	1:400	AB76442, Abcam	Goat anti-chicken Alexa fluor 488 IgG (H + L)	1:100
Choline Acetyltransferase polyclonal	Goat	1:20	AB144P; Millipore	Streptavidin 595 with biotinylated anti-goat IgG (H-L) made in rabbit	1:100 / 1:200
SOX-10 monoclonal	Rabbit	1:100	ab155279 Abcam	Donkey anti-rabbit Alexa-fluor 594 IgG (H + L)	1:100
GFAP monoclonal	Rabbit	1:200	345860 Merck Chemicals	Donkey anti-rabbit Alexa-fluor 594 IgG (H + L)	1:100
S100β monoclonal	Rabbit	1:200	ab52642 Abcam	Donkey anti-rabbit Alexa-fluor 594 IgG (H + L)	1:100
α-Synuclein monoclonal	Mouse	1:800	BD 610787 BD/Transduction	Donkey anti-mouse Alexa-fluor 594 IgG (H + L)	1:100

### Double staining

Prior to immunostaining, whole-mount samples were permeabilised for 1 h at room temperature in blocking buffer (10% normal goat or donkey serum, 5–10% PBS and 1% Triton X-100) to reduce non-specific binding. Whole-mounts were then incubated overnight in mixtures of primary antibodies diluted in the blocking buffer, at 4 °C. Anti- HuC/HuD antibody was used to identify all myenteric neuronal cell bodies^48^ (see Table 2). Whole-mounts were then washed with PBS and incubated with secondary antibodies diluted in the blocking buffer for 3 h at room temperature. Next, tissues were washed 3 × 10 min in PBS and then mounted on slides using Mowiol mounting media (final concentrations: Mowiol 40-88 (8%), glycerol (25%), DABCO (1%), sodium azide (0.1%) in Tris buffer 0.08 M pH 8.5).

### Data analyses and statistics

For comparison of basal resistance and short circuit currents (*I*_sc_), multiple readings from different animal groups (control and MPTP) were obtained. The overall mean ± SEM was determined from the individual average values of all animals in their respective groups, each *n* representing a single animal observation. For the amplitude and the frequency of spontaneous contractions, the values for the replicates running in parallel (*n* = 2–3) were averaged for each animal and presented as *n* = 1. Thus each *n* number represents a mean of multiple observations in each animal (so that for *n* = 8 there were between 18 and 24 observations). Data from the control and MPTP animals were compared using an unpaired, two-tailed Student’s *t*-test. Where multiple groups of data in concentration response curves were compared, a two-tailed, repeated measure two-way ANOVA followed by Fisher’s LSD multiple comparison post hoc test was used. For comparison of the effects of drugs on the *I*_sc_, a one-way ANOVA followed by Dunnett’s multiple comparison was carried out, where the effect of drugs on *I*_sc_ was compared to the effect of *I*_sc_ in dH_2_O. For comparison of cell counts and immunohistochemical analyses expressed as total counts/mm^2^ were means expressed as mean ± SEM and the differences between naive controls and MPTP samples were compared using an unpaired, two-tailed Student’s *t*-test. All statistical analyses were carried out using Prism 7.0 software (GraphPad, San Diego, CA, USA). Differences were considered statistically significant at *P* < 0.05.

### Reporting summary

Further information on research design is available in the Nature Research Reporting Summary linked to this article.

## Supplementary information

Reporting Summary.

## Data Availability

All data and figures will be available upon request.
