# Supplementary material for: Dysregulation of epithelial ion transport and neurochemical changes in the colon of a parkinsonian primate
Source: NPJ Parkinsons Dis. 2021 Jan 21;7:9. doi: 10.1038/s41531-020-00150-x (PMC7820491; doi:10.1038/s41531-020-00150-x)
Supplement: Supplementary file 1 — Reporting Summary. [file 41531_2020_150_MOESM1_ESM.pdf]

nature research

Corresponding author(s): Dr. Mahmoud Iravani

Reporting Summary

Nature Research wishes to improve the reproducibility of the work that we publish. This form provides structure for consistency and transparency in reporting. For further information on Nature Research policies, see Authors & Referees and the Editorial Policy Checklist.

Statistical parameters

When statistical analyses are reported, confirm that the following items are present in the relevant location (e.g. figure legend, table legend, main text, or Methods section).

n/a	Confirmed	
☐	☒	The exact sample size (*n*) for each experimental group/condition, given as a discrete number and unit of measurement
☐	☒	An indication of whether measurements were taken from distinct samples or whether the same sample was measured repeatedly
☐	☒	The statistical test(s) used AND whether they are one- or two-sided<br>*Only common tests should be described solely by name; describe more complex techniques in the Methods section.*
☒	☐	A description of all covariates tested
☒	☐	A description of any assumptions or corrections, such as tests of normality and adjustment for multiple comparisons
☐	☒	A full description of the statistics including central tendency (e.g. means) or other basic estimates (e.g. regression coefficient) AND variation (e.g. standard deviation) or associated estimates of uncertainty (e.g. confidence intervals)
☐	☒	For null hypothesis testing, the test statistic (e.g. *F*, *t*, *r*) with confidence intervals, effect sizes, degrees of freedom and *P* value noted<br>*Give P values as exact values whenever suitable.*
☒	☐	For Bayesian analysis, information on the choice of priors and Markov chain Monte Carlo settings
☒	☐	For hierarchical and complex designs, identification of the appropriate level for tests and full reporting of outcomes
☒	☐	Estimates of effect sizes (e.g. Cohen's *d*, Pearson's *r*), indicating how they were calculated
☐	☒	Clearly defined error bars<br>*State explicitly what error bars represent (e.g. SD, SE, CI)*

Our web collection on statistics for biologists may be useful.

Software and code

Policy information about availability of computer code

Data collection	All data for cell counts and short cuircuit currents were manually collected by multiple observers. The data generated by the contractile response of the colon was recorded using a force displacement transducer connected to a computerized data acquisition system (Lab-Chart AD Systems.
Data analysis	All statistical analyses were carried out using Prism 7.0 software (GraphPad, San Diego, CA USA). Differences were considered statistically significant at P < 0.05.

For manuscripts utilizing custom algorithms or software that are central to the research but not yet described in published literature, software must be made available to editors/reviewers upon request. We strongly encourage code deposition in a community repository (e.g. GitHub). See the Nature Research guidelines for submitting code & software for further information.

Data

Policy information about availability of data

All manuscripts must include a data availability statement. This statement should provide the following information, where applicable:

- Accession codes, unique identifiers, or web links for publicly available datasets
- A list of figures that have associated raw data
- A description of any restrictions on data availability

Provide your data availability statement here.

Field-specific reporting

Please select the best fit for your research. If you are not sure, read the appropriate sections before making your selection.

☒ Life sciences ☐ Behavioural & social sciences

For a reference copy of the document with all sections, see nature.com/authors/policies/ReportingSummary-flat.pdf

Life sciences

Study design

All studies must disclose on these points even when the disclosure is negative.

Sample size	There were a total of 15 adult common marmosets of either sex used. One group comprised of drug and toxin naïve controls (n=8).The second group (n=7 had been previously treated with MPTP 1.4 ± 0.4. The sample size was chosen based on availability of animals that upon sample size calculation would yield statistically meaningful data, if any statistically significant changes detected.
Data exclusions	For comparison of cell counts and immunohistochemical analyses expressed as total counts / mm2 were means expressed as mean ± s.e.m. and the differences between naïve controls and MPTP samples were compared using an un-paired Student's t-test. Tissues that were torn or damaged during processing were not included in analyses. If samples did not form an adequate seal in the Ussing chamber studies, they were rejected. Tissues from animal that did not contract >1g in response to 1µM carbachol were also rejected.
Replication	The overall mean ± s.e.m was determined from the individual average values of all animals in their respective groups. For calculation of spontaneous activity, 5 to 10 observations for each animal sample were meaned and this was treated as n=1 for that animals. For calculations of short circuit currents using Ussing chamber, each n represents an individual animal sample.
Randomization	The number of animals belonging to naive or MPTP were selected from a known pool number. On each day of experiments, only one animal was used in a semi-alternate manner. The animals background, weight, gender and prior pharmacological treatment were not taken into account when selecting each animal. Therefore, the animals were randomly selected.
Blinding	At the time of sample preparation the experimenter was blinded to the prior treatment of the animals. Samples were identified at the end of the study before data were placed in either the naive or MPTP treated group.

Materials & experimental systems

Policy information about availability of materials

n/a	Involved in the study
☒	☐ Unique materials
☐	☒ Antibodies
☒	☐ Eukaryotic cell lines
☐	☒ Research animals
☒	☐ Human research participants

Antibodies

Antibodies used	Neuronal protein, monoclonal - HuC/D Mouse 1:50 16A11:Molecular probes Donkey antimouse Alexafluor 350 IgG (H+L) 1:100<br>Vasoactive intestinal polypeptide polyclonal Rabbit 1:100 Sc-20727; Santa Cruz Biotechnology - Horse FITC anti-rabbit IgG (H+L) 1:100<br><br>Neuronal nitric oxide synthase polyclonal Rabbit 1:500 AB5380:Millipore Goat FITC anti-rabbit igG (H+L) 1:100<br>Tyrosine hydroxylase polyclonal Rabbit 1:500 P40101; Pel-Freez Biologicals, USA Biotinylated goat antirabbit-IgG (H+L) 1:200<br><br>Tyrosine hydroxylase polyclonal Chicken 1:400 AB76442, Abcam Goat antichicken Alexafluor 488 IgG (H+L) 1:100<br>Choline Acetyltransferase polyclonal Goat 1:20 AB144P; Millipore Streptavidin 595 with biotinylated anti-goat IgG (H-L) made in rabbit 1:100 /1:200

SOX-10 monoclonal Rabbit 1:100 ab155279 Abcam Donkey antirabbit Alexafluor 594 IgG (H+L) 1:100
GFAP monoclonal Rabbit 1:200 345860 Merck Chemicals Donkey antirabbit Alexafluor 594 IgG (H+L) 1:100

S100β monoclonal Rabbit 1:200 ab52642 Abcam Donkey antirabbit Alexafluor 594 IgG (H+L) 1:100

α-Synuclein monoclonal mouse 1:800 BD 610787 BD/Transduction Donkey antimouse Alexafluor 594 IgG (H+L) 1:100

Validation

Each antibody was validated using a negative control where the primary antibody was omitted. Data is also available for the use of the same antibodies in other publications.

Research animals

Policy information about studies involving animals; ARRIVE guidelines recommended for reporting animal research

Animals/animal-derived materials

All experiments were conducted in adult common marmosets of either sex.

Method-specific reporting

n/a	Involved in the study
☒	☐ ChIP-seq
☒	☐ Flow cytometry
☒	☐ Magnetic resonance imaging
